# Neutrophil Function in an Inflammatory Milieu of Rheumatoid Arthritis

**DOI:** 10.1155/2018/8549329

**Published:** 2018-12-03

**Authors:** Weiqian Chen, Qin Wang, Yini Ke, Jin Lin

**Affiliations:** Division of Rheumatology, First Affiliated Hospital, College of Medicine, Zhejiang University, Hangzhou, Zhejiang, China

## Abstract

Rheumatoid arthritis (RA) is an inflammatory autoimmune disease characterized by the presence of autoantibodies against citrullinated protein antigens and proinflammatory cytokines which cause chronic synovitis, bone erosion, and eventual deformity; however, the precise etiology of RA is unclear. In the early stage of RA, neutrophils migrate into the articular cavity, become activated, and exert their function in an inflammatory process, suggesting an essential role of neutrophils in the initial events contributing to the pathogenesis of RA. Solid evidence exists that supports the contribution of neutrophil extracellular traps (NETs) to the production of autoantibodies against citrullinated proteins which can trigger the immune reaction in RA. Concurrently, proinflammatory cytokines regulate the neutrophil migration, apoptosis, and NET formation. As a result, the inflammatory neutrophils produce more cytokines and influence other immune cells thereby perpetuating the inflammatory condition in RA. In this review, we summarize the advances made in improving our understanding of neutrophil migration, apoptosis, and NET formation in the presence of an RA inflammatory milieu. We will also discuss the most recent strategies in modulating the inflammatory microenvironment that have an impact on neutrophil function which may provide alternative novel therapies for RA.

## 1. Introduction

Rheumatoid arthritis (RA) is an autoimmune disease characterized by the presence of autoantibodies against citrullinated protein antigens (ACPAs) and proinflammatory cytokines which cause chronic synovitis, bone erosion, and eventual deformity. In the early stage of RA, there is a large infiltrate of neutrophils to the articular cavity, accumulating in both synovial tissue and fluid [1]. The presence of neutrophils in the synovial area correlates with the early clinical manifestations of joint inflammation, suggesting that neutrophils play a significant role in the initiation of RA [2]. Neutrophils are the first cells attracted by chemotactic cytokines and thus are quickly recruited into sites of infection or inflammation. As a member of the phagocytic innate immune system, neutrophils play an indispensable role during infection, injury, autoimmunity, and chronic disease. Neutrophils within circulating blood are captured by adhesion molecules on the endothelial surface of blood vessels and migrate from the bloodstream into pathological sites where invading pathogens are recognized by the host [3]. Neutrophils fulfill their protective functions through phagocytosis and release of granular enzymes and reactive oxygen species (ROS) as well as producing web-like structures called neutrophil extracellular traps (NETs) [4]. The release of granular enzymes and ROS into the extracellular space can cause damage to host tissues during inflammation [5]. Effective constitutive apoptosis of these cells is required for the resolution of inflammation. NETosis is a special type of neutrophil death which can produce NETs composed of a network of extracellular DNA fibers, histone proteins, elastase, and myeloperoxidase. NETs help neutrophils to immobilize and ensnare bacteria, fungi, or viruses, which results in a more effective elimination of these pathogens. It is reported that RA synovial fluid neutrophils show increased NETosis. Neutrophils from patients with RA are preactivated by immune complexes such as rheumatoid factor (RF), resulting in excessive ROS release, degranulation, and NETosis ex vivo [6]. Thus, the activation of neutrophils is associated with the initial inflammation in RA.

In addition, NETs can serve as a potential major source of citrullinated autoantigens which can trigger the development of autoimmune disease such as RA [7]. In recent years, studies have found that the formation of NETs is associated with autoantigens being detected in patients with RA. NET formation may drive ACPA production in the lung and promote the development of the early stage of RA [8]. Furthermore, it is reported that increased sputum NET levels are related to several citrullinated antibody reactivities in patients with RA [9]. Citrullinated autoantigens contained in NETs can be taken up by fibroblast-like synoviocytes (FLS) and presented to T cells in a major histocompatibility complex (MHC) II-dependent manner, leading to amplified antigen-specific T and B cell responses in RA [10]. Therefore, neutrophils may act as a bridge connecting FLS to T cell-mediated responses. In RA, the hallmark profile is the markedly increased proinflammatory cytokines which may regulate the neutrophil functions such as migration, apoptosis, and the formation of NETs [11–13]. In addition, activated neutrophils in the inflamed tissue can express a wide variety of proinflammatory cytokines and chemokines, which are also important to RA pathogenesis and neutrophils themselves [14]. For example, neutrophil-derived production of IL-8 (also known as CXCL8) leads to further rounds of neutrophil migration from the circulation, which enhances the inflammatory process [15]. In this review, we summarize the advances made in improving our understanding of neutrophil migration, apoptosis, and NET formation in the presence of an RA inflammatory milieu. We will also discuss the most recent strategies in targeting cytokines to modulate the function of neutrophils which may provide alternative novel therapies for RA.

## 2. Neutrophil Migration in RA

Neutrophils are major components in the synovial fluid of RA patients making up to 90% of cells. These cells are also abundant at the junction of the pannus and cartilage, where invasion occurs [16]. During the past decades, it has been reported that neutrophil depletion can greatly inhibit the development of arthritis in two different murine models, collagen antibody-induced arthritis (AIA) and the K/BxN models [2, 17]. Therefore, it is suggested that neutrophils play a crucial role in initiating the inflammatory response and the progression of arthritis in RA. It is important to know which cytokines can affect the neutrophil migration. By blocking or weakening the migration of neutrophil into the inflamed tissue, one would predict that this would lead to a decrease in disease activity of RA, thereby developing a new strategy for the treatment of RA.

It is well accepted that factors including cytokines, chemokines, and immune complexes (RF and ACPA) can contribute to the process of neutrophil migration. In this review, we focus on the cytokines that can orchestrate neutrophil migration in human RA and animal arthritis models. In RA, cytokines such as tumor necrosis factor alpha (TNF-*α*), interleukin-6 (IL-6), IL-17a, IL-22, IL-23, IL-1*β*, IL-8, interferon-*γ* (IFN-*γ*), granulocyte/macrophage colony-stimulating factor (GM-CSF), granulocyte colony-stimulating factor (G-CSF), IL-15, IL-18, IL-33, and IL-37 [11, 13, 18–36] are all detected either in the serum or in the synovial fluids of these patients. Thus, we discuss the impact of these cytokines on neutrophil migration in RA separately (Table 1).

### 2.1. TNF-*α* Enhances the Neutrophil Migration in RA

It is well known that TNF-*α* and IL-6 are two of the most predominant proinflammatory cytokines involved in the pathogenesis of RA. Anti-TNF-*α* and anti-IL-6R agents have been proven to be clinically useful in the disease control of RA. It is reported that TNF-*α* and IL-6 can both enhance the RA neutrophil migration *in vitro* [11, 21]. An additional study also finds that treatment with an anti-TNF-*α* agent can decrease the migratory capacity of neutrophils in patients with RA [18]. However, anti-IL-6R agents do not appear to affect neutrophil migration *in vitro* [21]. It indicates that TNF-*α* is a strong cytokine which can enhance RA neutrophil migration.

### 2.2. Th17-Related Cytokines Increase Neutrophil Migration in RA

Th17 cells (IL-17 and IL-22) and neutrophils are frequently observed in the synovial fluid of RA patients. A crosstalk exists between Th17 cells and neutrophils. Activated Th17 cells can directly attract neutrophils via the release of IL-8 [37]. Moreover, IL-17 and IL-23 can both induce neutrophil migration after intra-articular injection in a dose-dependent manner. In antigen-induced arthritis, early release of IL-23 can stimulate IL-17 production, in turn, causing a release of TNF-*α*, a variety of chemokines, and leukotriene B4 (LTB4), which together contribute to neutrophil recruitment. Therefore, IL-23/IL-17-induced neutrophil migration plays an important role in the pathogenesis of a murine model of RA [22]. Furthermore, injection of adenoviral vectors with IL-17 (AdIL-17) into the knee joints before induction of immune complex- (IC-) mediated arthritis has been shown to induce greater neutrophils migrating to the cartilage surface, exacerbating inflammation and cartilage destruction [38]. Interestingly, another Th17-related cytokine IL-22 is highly expressed in the synovial tissue of an antigen-induced arthritis mouse and human RA [25]. Furthermore, IL-22 inhibition and IL-22 genetic deficiency can reduce neutrophil migration in arthritic mice and improve articular pain [25]. Therefore, IL-17 and IL-22 both can promote the neutrophil migrating to the cartilage surface, exacerbating joint inflammation of RA.

### 2.3. IL-8 Promotes the Neutrophil Migration in RA

IL-8, a potent chemoattractant for neutrophils, plays a vital role in the recruitment and activation of neutrophils. IL-8 is considered to be one of the most crucial inflammatory chemokines involved in the development of arthritis [12]. Injection of lipopolysaccharide (LPS) or IL-1*α* into joints of rabbits induces arthritis accompanied by upregulation of IL-8. Blockade of IL-8 by neutralizing antibody can ameliorate arthritis and reduce the infiltration of neutrophils into the joints in the early phase of inflammation [39]. In RA patients, IL-8 is significantly elevated in the ACPA^high^ synovial fluid. The elevated level of IL-8 is associated with worse clinical manifestations of the disease. There is a positive correlation between IL-8 and the number of neutrophils in synovial fluid [40]. So IL-8 enhances the neutrophil migration and promotes the inflammatory response in RA.

### 2.4. IFN-*γ* Biphasically Affects Neutrophil Migration, While IL-15 Enhances the Neutrophil Migration in RA

IFN-*γ* has a dual function in the regulation of neutrophil migration. Firstly, IFN-*γ* is generally considered to be a proinflammatory factor produced by Th1 cells and natural killer cells. IFN-*γ* modulates neutrophil migration, as shown by a decrease of neutrophil recruitment in IFN-*γ*-deficient mice and recovery after reconstitution of the IFN-*γ* signaling pathway [41]. IFN-*γ*-induced neutrophil migration *in vivo* may be mediated by the release of the neutrophil chemotactic factor from resident macrophages [42]. In contrast, IFN-*γ* can also exert an anti-inflammatory effect in an RA animal model [43]. In the murine AIA model, the arthritis symptoms are exacerbated by the absence of IFN-*γ*. Furthermore, CXC chemokine receptor 2 (CXCR2)^+^ neutrophil recruitment is significantly higher in the joints of IFN-*γ*-deficient mice. IFN-*γ* receptor knockout (IFN-*γ*R KO) mice develop collagen-induced arthritis (CIA) more severely with an increased neutrophil influx, while anti-IL-17 antibody administration can ameliorate arthritis partly by reducing the neutrophil infiltration [44]. Based on these findings, it appears that there is a crosstalk between IFN-*γ* and IL-17 in inflammation. Meanwhile, IFN-*γ* modulates TNF-*α*-driven chemokine syntheses in RAFLS, resulting in a downregulation of IL-8 production [43]. IL-15 is a proinflammatory cytokine that has a wide variety of functions in autoimmune diseases. IL-15 has been found to be highly expressed in the synovial fluid of RA patients [34]. IL-15 can mediate blood and synovial neutrophil migration by triggering LTB4 production in antigen-induced arthritis models [45]. Furthermore, IL-15 can also induce NF-*κ*B activation and IL-8 production directly in human neutrophils, thereby activating these cells [46]. In a summary, IFN-*γ* bilaterally affects neutrophil migration, while IL-15 enhances the migration of neutrophil into joint.

### 2.5. GM-CSF and G-CSF Both Enhance the Neutrophil Migration in RA

GM-CSF and G-CSF are two hematopoietic growth factors involved in the regulation of hematopoiesis, used to treat neutropenia and to elicit the release of the hematopoietic stem cells from the bone marrow for transplantation [47]. They are both found in human RA serum and synovial fluid [29–31]. GM-CSF can activate and sustain the viability of neutrophils. GM-CSF^−/−^ mice are resistant to K/BxN serum transfer arthritis, whereas blockade of GM-CSF can ameliorate the severity of arthritis in the CIA mouse model with a simultaneous reduction in synovial neutrophils [48]. Recently, two clinical studies demonstrate that a monoclonal antibody targeting the GM-CSF receptor *α* called mavrilimumab is effective in the treatment of RA [49, 50]. However, the effect of this novel agent on neutrophil function has not yet been reported. Interestingly, G-CSF and G-CSF receptor-deficient mice are significantly protected in RA animal models partly due to a reduction in the number of neutrophils [47, 51]. A neutralizing antibody to the G-CSF receptor also prevents the progression of the disease by reducing neutrophil accumulation in the joints and inhibiting STAT3 phosphorylation in collagen Ab-induced arthritis [47]. Furthermore, the production of proinflammatory cytokines and chemokines is decreased in anti-G-CSF receptor-treated mice [47]. As such, targeting either GM-CSF or G-CSF may be a promising novel approach to treat RA via the downregulation of neutrophil migration.

### 2.6. IL-1 Family Cytokines Affect Neutrophil Migration in RA

IL-1 family is a group of 11 cytokines that play a central role in the regulation of immune responses. IL-1*β* is the most studied member because it is discovered first and it has a strongly proinflammatory effect. Importantly, IL-1*β* can promote neutrophil migration [25]. This is likely to contribute to RA disease progression since the level of IL-1*β* in the joint increases with the onset of arthritis and correlates highly with arthritis scores. Moreover, recombinant IL-1*β* (rIL-1*β*) administration into mice can induce arthritis, whereas arthritis development is arrested in IL-1R^−/−^ mice [52]. IL-18, another member of the IL-1 family, functions by binding to its receptor to induce IFN-*γ* production by T cells which can cause a rapid activation of the monocytes and macrophages. Recently, IL-18 is thought to be a potent mediator of inflammation in RA. It has been reported that IL-18 is significantly elevated in sera, synovial tissues, and synovial fluid of RA patients. IL-18 contributes to the inflammatory process by recruiting monocytes, lymphocytes, and neutrophils to the inflamed joints of RA [35]. In the CIA model, IL-18 activates neutrophils through a synthesis of LTB4 in response to TNF-*α*, which in turn enhances neutrophil recruitment [53]. IL-33 and IL-37 are both new members of the IL-1 family of cytokines. In methylated bovine serum albumin- (mBSA-) immunized mice, enhanced local neutrophil infiltration is associated with an increase in IL-33 mRNA expression. When recombinant IL-33 is injected into the joints of mice, it induces local neutrophil infiltration which subsequently resulted in joint damage. Moreover, neutrophil migration is inhibited by systemic and local administration of soluble IL-33R [11]. IL-33-induced neutrophil migration is dependent on increased levels of CXCL1, CCL3, TNF-*α*, and IL-1*β* concentrations in the joint [11]. Unlike other cytokines, IL-37 is an anti-inflammatory cytokine which suppresses joint and systemic inflammation [54]. IL-37 has a protective effect in an arthritis mouse model by reducing cell influx and inhibiting joint inflammation [54]. In a short conclusion, IL-1 family members, IL-1*β*, IL-18, and IL-33, all can induce local neutrophil migration, but IL-37 has an opposite effect.

## 3. Neutrophil Survival in RA

As we know, neutrophils usually have a limited lifespan in the circulation and rapidly undergo constitutive apoptosis when exposed to a stimulator such as TNF-*α* or Fas [55]. Once recruited into sites of the inflammation, the activated neutrophils can encounter with a variety of proinflammatory cytokines in the microenvironment that may affect their function and longevity [56]. Many cytokines are generated during inflammation, and these can induce a similar “primed” phenotype in neutrophils affecting their ability to undergo apoptosis. While proinflammatory factors such as GM-CSF, IFN-*γ*, IL-9, and IL-15 can reduce the induction of apoptosis and extend the lifespan of the neutrophils from RA patients *in vitro*, both IL-8 and IL-18 show no effect on this phenotype [27, 28, 32, 57–59]. In contrast, TNF-*α* and IL-6 exert a biphasic effect on neutrophil apoptosis [21, 27, 60–63].

### 3.1. GM-CSF, IFN-*γ*, IL-9, and IL-15 Delay Apoptosis of RA Neutrophils, but IL-8 and IL-18 Have No Effect

There is strong evidence that supports the role of GM-CSF in prolonging the neutrophil survival and activity in RA *in vitro* [27, 28, 57]. Both GM-CSF and IL-15 inhibit spontaneous apoptosis by downregulating proapoptotic proteins Bax and caspase-3, but only GM-CSF can abolish immune complex-induced apoptosis [64]. Importantly, IL-15 and IFN-*γ* can delay the apoptosis of RA neutrophils *in vitro* [27, 58]. Recently, Th9 cell frequency has been shown to be higher in synovial fluid of RA patients. Synovial IL-9 can decrease the apoptosis by inducing the expression of the antiapoptotic protein, Mcl-1, and prolonging the survival of the neutrophils [32]. IL-1*β* can reduce the spontaneous apoptosis of neutrophils from healthy controls *in vitro*, but not RA neutrophils [62, 65]. IL-8 and IL-18 do not affect the apoptosis of RA neutrophils *in vitro* [27, 59]. In summary, GM-CSF, IFN-*γ*, IL-9, and IL-15 can delay the apoptosis of neutrophils and extend their lifespan which may be harmful to inflammation resolution in RA [32].

### 3.2. TNF-*α* and IL-6 Exert a Biphasic Effect on Neutrophil Apoptosis

TNF-*α* shows an opposing effect where it enhances the rate of apoptosis in human neutrophils during the first 6 hours of exposure, followed by delayed apoptosis after 20 hours in culture [60, 61]. Furthermore, the regulation of TNF-*α* on human neutrophil apoptosis is dependent on the concentrations of TNF-*α in vitro*. Higher concentrations of TNF-*α* (≥10 ng/mL) increase the rate of caspase-dependent turnover of the antiapoptotic protein Mcl-1, which controls neutrophil survival, thereby accelerating neutrophil apoptosis, whereas lower concentrations of TNF-*α* (≤1 ng/mL) can stimulate the expression of the antiapoptotic protein Bfl-1 leading to enhanced neutrophil survival [61]. In contrast, other studies show that TNF-*α* can reduce the spontaneous apoptosis in neutrophils from healthy individuals (at ≤1 ng/mL) and RA patients (at 10 ng/mL) *in vitro* [27, 62]. Actually, the concentrations of TNF-*α* in RA plasma (<100 pg/mL) are far below those required to increase neutrophil apoptosis [57]. Meanwhile, IL-6 can inhibit apoptosis of neutrophils from a healthy donor [62, 64, 66] and RA patients *in vitro* [27]. However, others find that IL-6 enhances apoptosis in human neutrophils *in vitro* [63]. Recent evidence reveals that IL-6 indeed does not affect the rate of neutrophil apoptosis in patients with RA [21]. It has been reported that RA patients receiving the anti-IL-6 receptor agent tocilizumab (TCZ) may have decreased neutrophil counts [67]. However, TCZ *in vitro* does not induce apoptosis or phagocytosis of neutrophils. Also, TCZ does not affect neutrophil functions in RA patients receiving TCZ treatment ex vivo [21].

The mechanisms underlying the effects of priming cytokines on neutrophil apoptosis have been partially elucidated. GM-CSF reduces the rate of neutrophil apoptosis through activation of ERK1/2 or PI-3K/Akt pathways [68, 69]. GM-CSF has also been shown to decrease mRNA levels of the proapoptotic protein Bad while increasing its phosphorylation [60]. An RNAseq analysis of human neutrophils primed by proinflammatory cytokines (GM-CSF and TNF-*α*) *in vitro* revealed altered levels of 58 transcripts implicated in the control of apoptosis [70]. Synovial fluid of patients with RA contains a variety of antiapoptotic and proapoptotic cytokines. The hypoxic environment within these joints can have beneficial effects on neutrophil survival. Compared to arterial blood, inflamed tissues are usually more hypoxic and acidic. Hypoxia causes delayed apoptosis of neutrophils in humans and mice. Additional studies have found that hypoxia-induced neutrophil survival is mediated by HIF-1*α*-dependent NF-*κ*B activity [71]. Other studies demonstrate neutrophils isolated from RA synovial fluid and blood undergo delayed apoptosis as shown by increased expression of neutrophil survival protein Mcl-1 and decreased levels of activated caspase-9 [56, 57, 72]. The delay of neutrophil apoptosis is restored to control levels after treatment with methotrexate [72]. Interestingly, Janus kinase inhibitor (JAKi) prevents GM-CSF- and IFN-*γ*-induced apoptosis delay in RA and healthy control neutrophils in a dose-dependent manner. Incubation with JAKi prevents chemotaxis of RA neutrophils towards IL-8 but does not increase the level of apoptosis *in vitro* [58]. Therefore, the RA neutrophils in synovial fluid and blood undergo delayed apoptosis due to the proinflammatory and hypoxic microenvironment. GM-CSF, IFN-*γ*, IL-9, and IL-15 delay apoptosis of RA neutrophils, but others have no effect or a biphasic effect on neutrophil apoptosis.

## 4. The Role of NETs in the Pathogenesis of RA

Solid evidence exists that supports the important role of NETs in the pathogenesis of RA [8, 9, 13, 20, 73]. NET formation has been identified as a link between the innate and adaptive immune responses in autoimmunity. NETs provide the immune system with access to enriched sources of citrullinated proteins and thereby representing an early event preceding epitope spreading. Enhanced NETs are observed in both the bloodstream and synovial fluid of RA patients compared to healthy controls and patients with osteoarthritis [13, 73]. However, not all NETs shape the immune response toward disease-specific autoantibodies. One hypothesis speculates that NETs triggered by smoking can be an initiating factor in the pathogenesis of RA [74]. These NETs may drive anti-ACPA production in the lung which can play an important role in the early stage of RA development [8]. Increasing NET levels in the sputum are found to be significantly correlated with several citrullinated and noncitrullinated antibody reactivities in patients with RA [9]. Sera and immunoglobulin fractions from RA patients with high levels of ACPA or RF significantly enhanced NETosis. *In vitro* incubation of neutrophils with ACPA^high^ synovial fluid results in increased ROS production and extracellular DNA release compared to neutrophils incubated with ACPA-negative synovial fluid [20].

The inflammatory cytokines such as TNF-*α*, IL-17a, IL-6, and IL-8 can induce NETs in RA neutrophils. Subsequently, NETs promote more cytokine production and persistent inflammation via activating FLS or macrophages [13, 73, 75–78]. The neutralizing antibodies to TNF-*α* or IL-17R can reduce RA serum-induced-NETs [13]. NETs are strong activators of inflammatory responses in RA synovial fibroblasts since they can significantly augment the inflammatory reactions of RA synovial fibroblasts by inducing the production of IL-6, IL-8, chemokines, and adhesion molecules [13]. IL-6 can induce NET formation in neutrophils isolated from healthy individuals [75]. In RA, IL-6 may induce B cell maturation into plasma cells that can produce ACPA, thereby indirectly promoting NET formation [73]. Furthermore, anti-TNF-*α* (Infliximab) and anti-IL6R drugs (TCZ) can reduce the production of NETs [73, 76]. Interestingly, IL-8 can affect neutrophils through two different mechanisms of actions in RA. IL-8 recruits neutrophils to the inflammatory site through chemotactic effects while simultaneously triggering NET formation [13, 77].

IFN-*γ* and complement-5a (C5a) stimulation results in the formation of NETs in mature but not in immature human neutrophil populations [79]. Additional cytokines such as IL-1*β* and IL-18 are both able to stimulate NETs in human neutrophils [80–83]. CD177 is exclusively expressed in neutrophils and can be upregulated during inflammation. CD177^+^ neutrophils produce NETs through IL-22 signaling [84]. IL-9 and IL-33 are involved in the pathogenesis of several autoimmune diseases, such as RA [11, 32, 33]. Recently, neutrophils isolated from patients with systemic sclerosis (SSc) express IL-9R and exposure of these neutrophils to rIL-9 can significantly induce NET formation [85]. IL-33 enhances noninfectious inflammation in the liver by amplifying NET formation [86]. However, the role of IFN-*γ*, IL-9, IL-1*β*, IL-18, IL-22, and IL-33 in the formation of NETs in RA remains to be studied.

## 5. Parodontitis, NETs, and Anticitrulline Response in RA

Citrullines are produced not only in the lung but also in the oral cavity. Periodontitis, an oral bacterial infection, is very common in RA [87]. Patients with periodontitis appear to have increased the risk for RA and patients with RA have increased the risk for periodontitis [62]. It has been reported that periodontitis is associated with anti-CCP antibody and RA disease activity [87]. Most importantly, citrullinated proteins can be detected in the periodontium in RA patients with periodontitis [88]. As we know, the formation of citrullinated proteins is catalyzed by peptidylarginine deiminase (PAD) enzymes. Porphyromonas gingivalis (P. gingivalis), an oral bacteria that is a crucial factor for periodontitis and express PAD and citrullinated enolase, can mediate citrullination of bacterial and host protein [89]. It has been shown that deletion of the bacterial PAD gene results in complete abrogation of protein citrullination [90]. Moreover, antibody titers to P. gingivalis are correlated with ACPA titres of RA patients [91]. Therefore, PAD from P. gingivalis may cause citrullinated protein formation, which may elicit an antibody response to these proteins potentially leading to ACPA formation. Although P. gingivalis does not seem to directly regulate NET generation in RA [92], it has been shown that P. gingivalis could induce human neutrophils to generate NETs *in vitro* [93]. Interestingly, a pilot case-control study has found that periodontal treatment markedly reduced the serum levels of NETs in patients with RA and periodontitis [94]. Furthermore, Konig et al. find another periodontal germ called Aggregatibacter actinomycetemcomitans (Aa) can induce hypercitrullination in host neutrophils through its leukotoxin A (LtxA). Moreover, LtxA induces neutrophil lysis and the extracellular release of hypercitrullinated proteins mimicking extracellular trap formation. It has been confirmed in patients with RA that serum anti-LtxA antibodies are associated with both anticitrullinated protein antibodies and rheumatoid factor [95].

## 6. Neutrophils and Their Anti-inflammatory Effects

NETs have not only proinflammatory effects, but also contribute to the resolution of inflammation. Neutrophils can resolve inflammatory processes via the annexin A1- (AnxA1-) mediated pathway [96]. Microvesicles (MVs), those extracellular particles within a size range between 50 nm and 1000 nm, can mediate cell to cell communication by transferring proteins and lipids to target cells. MVs also have a role in antigen presentation and activation of endosomal receptors such as Toll-like receptors. Recently, MVs have been considered a functional molecule with their potential for the treatment of autoimmune disease. Neutrophil-derived MVs are present at higher levels in synovial fluids of RA [97]. Neutrophil-derived MVs can protect cartilage degradation through the reduction of interleukin-8 and prostaglandin E2. In addition, it has been demonstrated that synovial MVs overexpress the anti-inflammatory protein annexin A1 (AnxA1). Furthermore, neutrophil-derived MV-associated AnxA1 interacts with its receptor, increasing transforming growth factor-*β* production by chondrocytes ultimately leading to cartilage protection [97].

## 7. Conclusion and Perspectives

**Figure 1:**
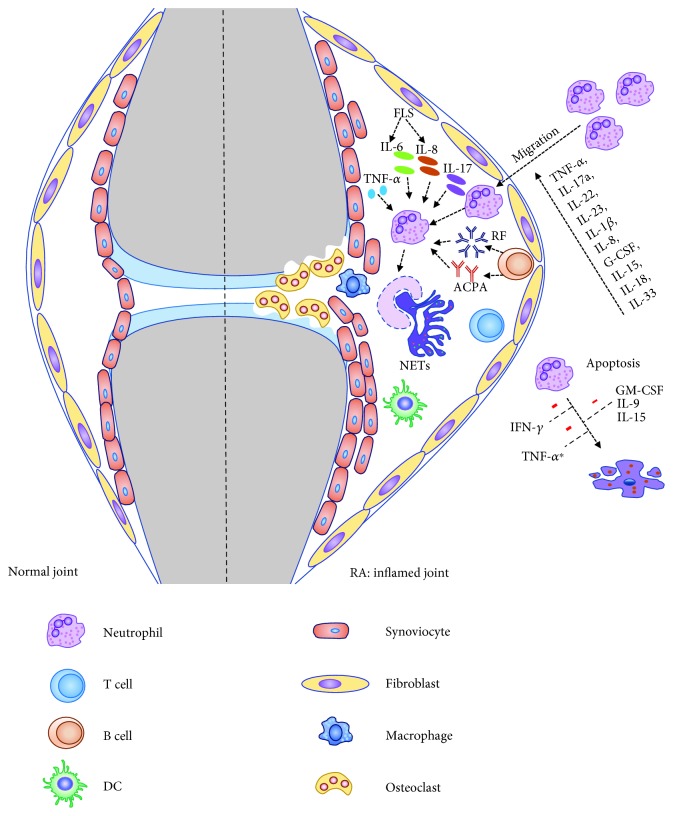
Role of neutrophil in the pathogenesis of RA. Proinflammatory cytokines in the joint can influence the migration of neutrophils. Neutrophils are activated by immune complexes and inflammatory cytokines (TNF-*α*, IL-6, IL-8, and IL-17a) within the synovial fluid, frequently causing enhanced NET formation in RA. In turn, NETs are served as a source of citrullinated autoantigens, further triggering the production of ACPA. Meanwhile, neutrophils undergo delayed apoptosis in an inflammatory milieu (GM-CSF, IL-9, IL-15, IFN-*γ*, and TNF-*α*) leading to persistent inflammation and joint damage in RA. ^∗^Controversial.

Neutrophils play a crucial role in initiating inflammatory response and progression of arthritis in RA (Figure 1). Cytokines which can regulate migration and activation of neutrophils are highly expressed in the inflamed joint tissues of RA patients. Proinflammatory factors such as GM-CSF, IFN-*γ*, IL-9, and IL-15 can reduce neutrophil apoptosis and extend their neutrophil lifespan in RA, whereas TNF-*α* and IL-6 display a biphasic effect on the apoptosis of neutrophils. NETs can be a source of released self-antigens found in RA. Formation of NETs is closely related to an autoantigen-triggered immune response in patients with RA. Furthermore, the inflammatory cytokines such as TNF-*α*, IL-6, IL-8, and IL-17a can induce NETs in RA neutrophils. Subsequently, NETs can promote an increase in cytokine production and enable the persistence of inflammation via the licensing of FLS and/or macrophages. Importantly, the anti-TNF-*α* and anti-IL-6R have been successful in treating RA by partially acting on neutrophils. Additional cytokines, for example, IL-22, IL-23, IL-18, G-CSF, IL-33, and IL-37, may also play an important role in the regulation of neutrophil functions. In addition, neutrophils may also play a role in the resolution of inflammation in RA. In the future, further studies are needed to understand how neutrophils act in an inflammatory milieu in patients with RA and animal models which may be critical for the development of novel treatment strategies.

## Figures and Tables

**Table 1:** Known cytokines' capacity to induce biological changes in neutrophils in RA.

Cytokines/blockade	Human RA or mouse model	Expression	Migration or recruitment	Apoptosis or survival	NET formation	Outcome
TNF-*α*	Human RA	↑ in serum and synovial fluid [13, 18, 19]	↑ *in vitro* [11]	Promote or delayed apoptosis *In vitro* [27]	↑ *in vitro* [13]	
Blockade of TNF-*α*	Human RA	—	↓i*n vivo* [18]	Antiapoptotic Mcl-1 ↑ and proapoptotic caspase-9 ↓ [57]	↓ *in vitro* [13, 73]	Improved [73, 98]
TNFR^−/−^	Collagen Ab and LPS-inducedarthritis model	—	↓ *in vivo* [52]	—	—	Improved [52]
IL-6	Human RA	↑ in serum and synovial fluid [19–21]	No effect *in vitro* [21]	No effect *in vitro* [21], delayed apoptosis *in vitro* [27]	↑ *in vitro* [73] ↑ *in vitro*^∗^ [75]	—
Blockade of IL-6	Human RA	—	—	No effect *in vivo* and ex vivo [21]	↓ *in vivo* and *in vitro* [73, 76]	Improved [99]
IL-17a	AIA mouse model and human RA	↑ in the joint [22, 23]	↑ *in vivo* [22]	—	↑ *in vitro* [13]	
Adenoviral vectors with IL-17	IC-mediated arthritis mouse model	—	↑ *in vivo* [38]	—	—	Deteriorated [38]
Anti-IL-17 antibody administration	CIA model	—	↓ *in vivo* [44]	—	↓ *in vitro* [13]	Improved [44]
IL-23	AIA mouse model and human RA	↑ in joint [22, 24]	↑ *in vivo* [22]	—	—	—
IL-22	AIA mouse model and human RA	↑ in synovial tissue [25, 26]	↑ *in vivo* [25]	—	—	—
Block IL-22 or IL-22^−/−^	AIA mouse model	—	↓ *in vivo* [25]	—	—	Improved [25]
rmIL-22 administration	Normal mice	—	↑ *in vivo* [25]	—	—	Deteriorated [25]
IL-1*β*	Human RA and AIA mouse model	↑ in synovial fluid [19]	↑ *in vitro* [25]	Delayed apoptosis *in vitro*^∗^ [62]	↑ *in vitro*^∗^ [81–83]	—
IL-1R^−/−^ or IL-1R antagonist	Collagen Ab and LPS-inducedarthritis model	—	↓ *in vivo* [52]	—	↓ *in vitro*^∗^ [82, 83]	Improved [52]
IL-8	Arthritis rabbit model and human RA	↑ in synovial fluid [19, 27]	↑ *in vivo* [39], ↑ *in vitro* [58]	No effect *in vitro* [27]	↑ *in vitro* [13]	—
Blockade of IL-8	Arthritis rabbit model	—	↓ *in vivo* [39]	—	—	Improved [39]
IL-10	Human RA	↑ in synovial fluid [19]	—	—	↑ *in vitro*^∗^ [100]	—
IFN-*γ*	Human RA	↑ in synovial fluid [19]	—	Delayed apoptosis *in vitro* [58]	↑ *in vitro*^∗^ [79]	—
IFN-*γ*^−/−^	AIA, CIA model	—	↑ *in vivo* [43, 44]	—	—	Deteriorated [43, 44]
GM-CSF	Human RA	↑ released by RAFLS *in vitro* [28], ↑ in serum and synovial fluid [18, 29, 30]	—	Survival ↑ [28], delayed apoptosis in vitro [27, 57, 58]	No effect *in vitro* [13]	—
Blockade of GM-CSF	CIA	—	↓ neutrophils in the joint [48]	—	—	Improved [48]
GM-CSF^−/−^	K/BxN serum transfer arthritis	—	↓ neutrophils in the joint [48]	—	—	Improved [48]
G-CSF	Human RA	↑ in serum and synovial fluid [30, 31]	—	—	↑ *in vitro*^∗^ [101]	—
rhG-CSF	CIA	↑ serum G-CSF [51]	↑ *in vivo* [51]	—	—	Deteriorated [51]
G-CSF^−/−^	CIA	—	↓ *in vivo* [51]	—	—	Improved [51]
mAb to G-CSF receptor	Collagen Ab-induced arthritis model	—	↓ *in vivo* [47]	—	—	Improved [47]
IL-9	Human RA	↑ in serum and synovial fluid [32, 33]	—	Delayed apoptosis [32]	↑ *in vitro*^∗^ [85]	—
IL-15	Human RA	↑ in synovial fluid [34]	↑ *in vivo* [45]	Delayed apoptosis *in vitro* [27]	↑ *in vitro*^∗^ [100]	
IL-18	Human RA	↑ in serum, synovial tissue and fluid [35]	↑ *in vivo* [53]	No effect *in vitro* [59]	↑ *in vitro*^∗^ [100]	
IL-33	mBSA-immunized mouse model	↑ mRNA expression [11]	↑ *in vivo* [11]	—	↑ *in vitro*^∗^ [86]	
rmIL-33	rmIL-33 local injection	—	↑ *in vivo* [11]	—	—	Deteriorated [11]
Blockade of IL-33	mBSA-immunized mouse model	—	↓ *in vivo* [11]	—	—	Improved [11]
IL-37	Human RA	↑ in serum and synovial fluid [36]	—	—	—	
IL-37 administration	CIA and streptococcal cell wall fragments induced arthritis	—	↓ *in vivo* [54]	—	—	Improved [54, 102]

RA: rheumatoid arthritis, NETs: neutrophil extracellular traps, TNF-*α*: tumor necrosis factor-alpha, Mcl-1: myeloid cell leukemia-1, TNFR: tumor necrosis factor receptor, Ab: antibody, LPS: lipopolysaccharide, IL: interleukin, AIA: antigen-induced arthritis, IC: immune complex, rmIL-22: recombinant murine interleukin 22, IL-1R: interleukin 1 receptor, IFN-*γ*: interferon gamma, CIA: collagen-induced arthritis, GM-CSF: granulocyte/macrophage colony-stimulating factor, RAFLS: rheumatoid arthritis synovial fibroblasts, G-CSF: granulocyte colony-stimulating factor, mAb: monoclonal antibody, mBSA: methylated bovine serum albumin, rmIL-33: recombinant murine interleukin 33, rhIL-37: recombinant human interleukin 37. ^∗^ is found in other inflammatory diseases or healthy individuals, but not RA

## References

[1] Marchi L. F., Paoliello-Paschoalato A. B., Oliveira R. D. R. (2018). Activation status of peripheral blood neutrophils and the complement system in adult rheumatoid arthritis patients undergoing combined therapy with infliximab and methotrexate. *Rheumatology International*.

[2] Tanaka D., Kagari T., Doi H., Shimozato T. (2006). Essential role of neutrophils in anti-type II collagen antibody and lipopolysaccharide-induced arthritis. *Immunology*.

[3] Nauseef W. M., Borregaard N. (2014). Neutrophils at work. *Nature Immunology*.

[4] Yang H., Biermann M. H., Brauner J. M., Liu Y., Zhao Y., Herrmann M. (2016). New insights into neutrophil extracellular traps: mechanisms of formation and role in inflammation. *Frontiers in Immunology*.

[5] Glennon-Alty L., Hackett A. P., Chapman E. A., Wright H. L. (2018). Neutrophils and redox stress in the pathogenesis of autoimmune disease. *Free Radical Biology & Medicine*.

[6] Corsiero E., Pratesi F., Prediletto E., Bombardieri M., Migliorini P. (2016). NETosis as source of autoantigens in rheumatoid arthritis. *Frontiers in Immunology*.

[7] Apel F., Zychlinsky A., Kenny E. F. (2018). The role of neutrophil extracellular traps in rheumatic diseases. *Nature Reviews Rheumatology*.

[8] Demoruelle M. K., Harrall K. K., Ho L. (2017). Anti-citrullinated protein antibodies are associated with neutrophil extracellular traps in the sputum in relatives of rheumatoid arthritis patients. *Arthritis & Rhematology*.

[9] Demoruelle M. K., Bowers E., Lahey L. J. (2018). Antibody responses to citrullinated and noncitrullinated antigens in the sputum of subjects with rheumatoid arthritis and subjects at risk for development of rheumatoid arthritis. *Arthritis & Rhematology*.

[10] Carmona-Rivera C., Carlucci P. M., Moore E. (2017). Synovial fibroblast-neutrophil interactions promote pathogenic adaptive immunity in rheumatoid arthritis. *Science Immunology*.

[11] Verri W. A., Souto F. O., Vieira S. M. (2010). IL-33 induces neutrophil migration in rheumatoid arthritis and is a target of anti-TNF therapy. *Annals of the Rheumatic Diseases*.

[12] Zhu X., Xiao L., Huo R. (2013). Cyr 61 is involved in neutrophil infiltration in joints by inducing IL-8 production by fibroblast-like synoviocytes in rheumatoid arthritis. *Arthritis Research & Therapy*.

[13] Khandpur R., Carmona-Rivera C., Vivekanandan-Giri A. (2013). NETs are a source of citrullinated autoantigens and stimulate inflammatory responses in rheumatoid arthritis. *Science Translational Medicine*.

[14] Mantovani A., Cassatella M. A., Costantini C., Jaillon S. (2011). Neutrophils in the activation and regulation of innate and adaptive immunity. *Nature Reviews Immunology*.

[15] Fujishima S., Hoffman A. R., Vu T. (1993). Regulation of neutrophil interleukin 8 gene expression and protein secretion by LPS, TNF-*α*, and IL-1*β*. *Journal of Cellular Physiology*.

[16] Weissmann G., Korchak H. (1984). Rheumatoid arthritis: the role of neutrophil activation. *Inflammation*.

[17] Wipke B. T., Allen P. M. (2001). Essential role of neutrophils in the initiation and progression of a murine model of rheumatoid arthritis. *The Journal of Immunology*.

[18] den Broeder A. A., Wanten G. J. A., Oyen W. J. G., Naber T., van Riel P. L. C. M., Barrera P. (2003). Neutrophil migration and production of reactive oxygen species during treatment with a fully human anti-tumor necrosis factor-alpha monoclonal antibody in patients with rheumatoid arthritis. *The Journal of Rheumatology*.

[19] Schlaak J. F., Pfers I., Meyer Zum Büschenfelde K. H., Märker-Hermann E. (1996). Different cytokine profiles in the synovial fluid of patients with osteoarthritis, rheumatoid arthritis and seronegative spondylarthropathies. *Clinical and Experimental Rheumatology*.

[20] Gorlino C. V., Dave M. N., Blas R. (2018). Association between levels of synovial anti-citrullinated peptide antibodies and neutrophil response in patients with rheumatoid arthritis. *European Journal of Immunology*.

[21] Wright H. L., Cross A. L., Edwards S. W., Moots R. J. (2014). Effects of IL-6 and IL-6 blockade on neutrophil function in vitro and in vivo. *Rheumatology*.

[22] Lemos H. P., Grespan R., Vieira S. M. (2009). Prostaglandin mediates IL-23/IL-17-induced neutrophil migration in inflammation by inhibiting IL-12 and IFN*γ* production. *Proceedings of the National Academy of Sciences of the United States of America*.

[23] van Baarsen L. G. M., Lebre M. C., van der Coelen D. (2014). Heterogeneous expression pattern of interleukin 17A (IL-17A), IL-17F and their receptors in synovium of rheumatoid arthritis, psoriatic arthritis and osteoarthritis: possible explanation for nonresponse to anti-IL-17 therapy?. *Arthritis Research & Therapy*.

[24] Kageyama Y., Ichikawa T., Nagafusa T., Torikai E., Shimazu M., Nagano A. (2007). Etanercept reduces the serum levels of interleukin-23 and macrophage inflammatory protein-3 alpha in patients with rheumatoid arthritis. *Rheumatology International*.

[25] Pinto L. G., Talbot J., Peres R. S. (2015). Joint production of IL-22 participates in the initial phase of antigen-induced arthritis through IL-1*β* production. *Arthritis Research & Therapy*.

[26] da Rocha L. F., Duarte Â. L. B. P., Dantas A. T. (2012). Increased serum interleukin 22 in patients with rheumatoid arthritis and correlation with disease activity. *The Journal of Rheumatology*.

[27] Ottonello L., Cutolo M., Frumento G. (2002). Synovial fluid from patients with rheumatoid arthritis inhibits neutrophil apoptosis: role of adenosine and proinflammatory cytokines. *Rheumatology*.

[28] Parsonage G., Filer A., Bik M. (2008). Prolonged, granulocyte-macrophage colony-stimulating factor-dependent, neutrophil survival following rheumatoid synovial fibroblast activation by IL-17 and TNFalpha. *Arthritis Research & Therapy*.

[29] Katano M., Okamoto K., Arito M. (2009). Implication of granulocyte-macrophage colony-stimulating factor induced neutrophil gelatinase-associated lipocalin in pathogenesis of rheumatoid arthritis revealed by proteome analysis. *Arthritis Research & Therapy*.

[30] Stabler T., Piette J. C., Chevalier X., Marini-Portugal A., Kraus V. B. (2004). Serum cytokine profiles in relapsing polychondritis suggest monocyte/macrophage activation. *Arthritis and Rheumatism*.

[31] Nakamura H., Ueki Y., Sakito S. (2000). High serum and synovial fluid granulocyte colony stimulating factor (G-CSF) concentrations in patients with rheumatoid arthritis. *Clinical and Experimental Rheumatology*.

[32] Chowdhury K., Kumar U., Das S. (2018). Synovial IL-9 facilitates neutrophil survival, function and differentiation of Th17 cells in rheumatoid arthritis. *Arthritis Research & Therapy*.

[33] Dantas A. T., Marques C. D. L., da Rocha Junior L. F. (2015). Increased serum interleukin-9 levels in rheumatoid arthritis and systemic lupus erythematosus: pathogenic role or just an epiphenomenon?. *Disease Markers*.

[34] McInnes I. B., al-Mughales J., Field M. (1996). The role of interleukin-15 in T-cell migration and activation in rheumatoid arthritis. *Nature Medicine*.

[35] Volin M. V., Koch A. E. (2011). Interleukin-18: a mediator of inflammation and angiogenesis in rheumatoid arthritis. *Journal of Interferon & Cytokine Research*.

[36] Xia L., Shen H., Lu J. (2015). Elevated serum and synovial fluid levels of interleukin-37 in patients with rheumatoid arthritis: attenuated the production of inflammatory cytokines. *Cytokine*.

[37] Pelletier M., Maggi L., Micheletti A. (2010). Evidence for a cross-talk between human neutrophils and Th17 cells. *Blood*.

[38] Grevers L. C., van Lent P. L. E. M., Koenders M. I. (2009). Different amplifying mechanisms of interleukin-17 and interferon-gamma in Fcgamma receptor-mediated cartilage destruction in murine immune complex-mediated arthritis. *Arthritis and Rheumatism*.

[39] Akahoshi T., Endo H., Kondo H. (1994). Essential involvement of interleukin-8 in neutrophil recruitment in rabbits with acute experimental arthritis induced by lipopolysaccharide and interleukin-1. *Lymphokine and Cytokine Research*.

[40] Peichl P., Ceska M., Effenberger F., Haberhauer G., Broell H., Lindley I. J. D. (1991). Presence of NAP-1/IL-8 in synovial fluids indicates a possible pathogenic role in rheumatoid arthritis. *Scandinavian Journal of Immunology*.

[41] McLoughlin R. M., Witowski J., Robson R. L. (2003). Interplay between IFN-*γ* and IL-6 signaling governs neutrophil trafficking and apoptosis during acute inflammation. *The Journal of Clinical Investigation*.

[42] Ribeiro R. A., Cunha F. Q., Ferreira S. H. (1990). Recombinant gamma interferon causes neutrophil migration mediated by the release of a macrophage neutrophil chemotactic factor. *International Journal of Experimental Pathology*.

[43] Williams A. S., Richards P. J., Thomas E. (2007). Interferon-gamma protects against the development of structural damage in experimental arthritis by regulating polymorphonuclear neutrophil influx into diseased joints. *Arthritis and Rheumatism*.

[44] Kelchtermans H., Schurgers E., Geboes L. (2009). Effector mechanisms of interleukin-17 in collagen-induced arthritis in the absence of interferon-*γ* and counteraction by interferon-*γ*. *Arthritis Research & Therapy*.

[45] Verri W. . A., Cunha T. . M., Ferreira S. . H. (2007). IL-15 mediates antigen-induced neutrophil migration by triggering IL-18 production. *European Journal of Immunology*.

[46] McDonald P. P., Russo M. P., Ferrini S., Cassatella M. A. (1998). Interleukin-15 (IL-15) induces NF-*κ*B activation and IL-8 production in human neutrophils. *Blood*.

[47] Campbell I. K., Leong D., Edwards K. M. (2016). Therapeutic targeting of the G-CSF receptor reduces neutrophil trafficking and joint inflammation in antibody-mediated inflammatory arthritis. *Journal of Immunology*.

[48] Cook A. D., Turner A. L., Braine E. L., Pobjoy J., Lenzo J. C., Hamilton J. A. (2011). Regulation of systemic and local myeloid cell subpopulations by bone marrow cell-derived granulocyte-macrophage colony-stimulating factor in experimental inflammatory arthritis. *Arthritis and Rheumatism*.

[49] Burmester G. R., McInnes I., Kremer J. (2017). A randomised phase IIb study of mavrilimumab, a novel GM-CSF receptor alpha monoclonal antibody, in the treatment of rheumatoid arthritis. *Annals of the Rheumatic Diseases*.

[50] Weinblatt M. E., McInnes I. B., Kremer J. M. (2018). A randomized phase IIb study of mavrilimumab and golimumab in rheumatoid arthritis. *Arthritis & Rhematology*.

[51] Eyles J. L., Hickey M. J., Norman M. U. (2008). A key role for G-CSF-induced neutrophil production and trafficking during inflammatory arthritis. *Blood*.

[52] Kagari T., Doi H., Shimozato T. (2002). The importance of IL-1*β* and TNF-*α*, and the noninvolvement of IL-6, in the development of monoclonal antibody-induced arthritis. *Journal of Immunology*.

[53] Cannetti C. A., Leung B. P., Culshaw S., McInnes I. B., Cunha F. Q., Liew F. Y. (2003). IL-18 enhances collagen-induced arthritis by recruiting neutrophils via TNF-*α* and leukotriene B_4_. *Journal of Immunology*.

[54] Cavalli G., Koenders M., Kalabokis V. (2016). Treating experimental arthritis with the innate immune inhibitor interleukin-37 reduces joint and systemic inflammation. *Rheumatology*.

[55] Murray J., Barbara J. A., Dunkley S. A. (1997). Regulation of neutrophil apoptosis by tumor necrosis factor-*α*: requirement for TNFR55 and TNFR75 for induction of apoptosis in vitro. *Blood*.

[56] Cross A., Barnes T., Bucknall R. C., Edwards S. W., Moots R. J. (2006). Neutrophil apoptosis in rheumatoid arthritis is regulated by local oxygen tensions within joints. *Journal of Leukocyte Biology*.

[57] Wright H. L., Chikura B., Bucknall R. C., Moots R. J., Edwards S. W. (2011). Changes in expression of membrane TNF, NF-*ĸ*B activation and neutrophil apoptosis during active and resolved inflammation. *Annals of the Rheumatic Diseases*.

[58] Mitchell T. S., Moots R. J., Wright H. L. (2017). Janus kinase inhibitors prevent migration of rheumatoid arthritis neutrophils towards interleukin-8, but do not inhibit priming of the respiratory burst or reactive oxygen species production. *Clinical and Experimental Immunology*.

[59] Leung B. P., Culshaw S., Gracie J. A. (2001). A role for IL-18 in neutrophil activation. *Journal of Immunology*.

[60] Cowburn A. S., Cadwallader K. A., Reed B. J., Farahi N., Chilvers E. R. (2002). Role of PI3-kinase-dependent Bad phosphorylation and altered transcription in cytokine-mediated neutrophil survival. *Blood*.

[61] Cross A., Moots R. J., Edwards S. W. (2008). The dual effects of TNF*α* on neutrophil apoptosis are mediated via differential effects on expression of Mcl-1 and Bfl-1. *Blood*.

[62] McNamee J. P., Bellier P. V., Kutzner B. C., Wilkins R. C. (2005). Effect of pro-inflammatory cytokines on spontaneous apoptosis in leukocyte sub-sets within a whole blood culture. *Cytokine*.

[63] Afford S. C., Pongracz J., Stockley R. A., Crocker J., Burnett D. (1992). The induction by human interleukin-6 of apoptosis in the promonocytic cell line U937 and human neutrophils. *The Journal of Biological Chemistry*.

[64] Ottonello L., Frumento G., Arduino N. (2002). Differential regulation of spontaneous and immune complex-induced neutrophil apoptosis by proinflammatory cytokines. Role of oxidants, Bax and caspase-3. *Journal of Leukocyte Biology*.

[65] Christenson K., Björkman L., Karlsson A., Bylund J. (2013). Regulation of neutrophil apoptosis differs after in vivo transmigration to skin chambers and synovial fluid: a role for inflammasome-dependent interleukin-1*β* release. *Journal of Innate Immunity*.

[66] Biffl W. L., Moore E. E., Moore F. A., Barnett C. C. (1995). Interleukin-6 suppression of neutrophil apoptosis is neutrophil concentration dependent. *Journal of Leukocyte Biology*.

[67] Moots R. J., Sebba A., Rigby W. (2017). Effect of tocilizumab on neutrophils in adult patients with rheumatoid arthritis: pooled analysis of data from phase 3 and 4 clinical trials. *Rheumatology*.

[68] Klein J. B., Buridi A., Coxon P. Y. (2001). Role of extracellular signal-regulated kinase and phosphatidylinositol-3 kinase in chemoattractant and LPS delay of constitutive neutrophil apoptosis. *Cellular Signalling*.

[69] Klein J. B., Rane M. J., Scherzer J. A. (2000). Granulocyte-macrophage colony-stimulating factor delays neutrophil constitutive apoptosis through phosphoinositide 3-kinase and extracellular signal-regulated kinase pathways. *Journal of Immunology*.

[70] Wright H. L., Thomas H. B., Moots R. J., Edwards S. W. (2013). RNA-seq reveals activation of both common and cytokine-specific pathways following neutrophil priming. *PLoS One*.

[71] Walmsley S. R., Print C., Farahi N. (2005). Hypoxia-induced neutrophil survival is mediated by HIF-1*α*-dependent NF-*κ*B activity. *The Journal of Experimental Medicine*.

[72] Weinmann P., Moura R. A., Caetano-Lopes J. R. (2007). Delayed neutrophil apoptosis in very early rheumatoid arthritis patients is abrogated by methotrexate therapy. *Clinical and Experimental Rheumatology*.

[73] Perez-Sanchez C., Ruiz-Limon P., Aguirre M. A. (2017). Diagnostic potential of NETosis-derived products for disease activity, atherosclerosis and therapeutic effectiveness in rheumatoid arthritis patients. *Journal of Autoimmunity*.

[74] Skopelja-Gardner S., Jones J. D., Rigby W. F. C. (2018). "NETtling" the host: breaking of tolerance in chronic inflammation and chronic infection. *Journal of Autoimmunity*.

[75] Joshi M. B., Lad A., Bharath Prasad A. S., Balakrishnan A., Ramachandra L., Satyamoorthy K. (2013). High glucose modulates IL-6 mediated immune homeostasis through impeding neutrophil extracellular trap formation. *FEBS Letters*.

[76] Ruiz-Limón P., Ortega R., Arias de la Rosa I. (2017). Tocilizumab improves the proatherothrombotic profile of rheumatoid arthritis patients modulating endothelial dysfunction, NETosis, and inflammation. *Translational Research*.

[77] Gupta A. K., Hasler P., Holzgreve W., Gebhardt S., Hahn S. (2005). Induction of neutrophil extracellular DNA lattices by placental microparticles and IL-8 and their presence in preeclampsia. *Human Immunology*.

[78] Warnatsch A., Ioannou M., Wang Q., Papayannopoulos V. (2015). Neutrophil extracellular traps license macrophages for cytokine production in atherosclerosis. *Science*.

[79] Martinelli S., Urosevic M., Daryadel A. (2004). Induction of genes mediating interferon-dependent extracellular trap formation during neutrophil differentiation. *The Journal of Biological Chemistry*.

[80] Kahlenberg J. M., Carmona-Rivera C., Smith C. K., Kaplan M. J. (2013). Neutrophil extracellular trap-associated protein activation of the NLRP3 inflammasome is enhanced in lupus macrophages. *Journal of Immunology*.

[81] Keshari R. S., Jyoti A., Dubey M. (2012). Cytokines induced neutrophil extracellular traps formation: implication for the inflammatory disease condition. *PLoS One*.

[82] Meher A. K., Spinosa M., Davis J. P. (2018). Novel role of IL (interleukin)-1*β* in neutrophil extracellular trap formation and abdominal aortic aneurysms. *Arteriosclerosis, Thrombosis, and Vascular Biology*.

[83] Mitroulis I., Kambas K., Chrysanthopoulou A. (2011). Neutrophil extracellular trap formation is associated with IL-1*β* and autophagy-related signaling in gout. *PLoS One*.

[84] Zhou G., Yu L., Fang L. (2018). CD177^+^ neutrophils as functionally activated neutrophils negatively regulate IBD. *Gut*.

[85] Guggino G., Lo Pizzo M., Di Liberto D. (2017). Interleukin-9 over-expression and T helper 9 polarization in systemic sclerosis patients. *Clinical and Experimental Immunology*.

[86] Yazdani H. O., Chen H.-W., Tohme S. (2018). IL-33 exacerbates liver sterile inflammation by amplifying neutrophil extracellular trap formation. *Journal of Hepatology*.

[87] Mikuls T. R., Payne J. B., Yu F. (2014). Periodontitis and Porphyromonas gingivalis in patients with rheumatoid arthritis. *Arthritis & Rhematology*.

[88] Nesse W., Westra J., Wal J. E. (2012). The periodontium of periodontitis patients contains citrullinated proteins which may play a role in ACPA (anti-citrullinated protein antibody) formation. *Journal of Clinical Periodontology*.

[89] Vitkov L., Hartl D., Minnich B., Hannig M. (2017). Janus-faced neutrophil extracellular traps in periodontitis. *Frontiers in Immunology*.

[90] Wegner N., Wait R., Sroka A. (2010). Peptidylarginine deiminase from Porphyromonas gingivalis citrullinates human fibrinogen and *α*-enolase: implications for autoimmunity in rheumatoid arthritis. *Arthritis and Rheumatism*.

[91] Mikuls T. R., Payne J. B., Reinhardt R. A. (2009). Antibody responses to *Porphyromonas gingivalis* (*P. gingivalis*) in subjects with rheumatoid arthritis and periodontitis. *International Immunopharmacology*.

[92] Jayaprakash K., Demirel I., Khalaf H., Bengtsson T. (2015). The role of phagocytosis, oxidative burst and neutrophil extracellular traps in the interaction between neutrophils and the periodontal pathogen Porphyromonas gingivalis. *Molecular Oral Microbiology*.

[93] Delbosc S., Alsac J. M., Journe C. (2011). *Porphyromonas gingivalis* participates in pathogenesis of human abdominal aortic aneurysm by neutrophil activation. Proof of concept in rats. *PLoS One*.

[94] Kaneko C., Kobayashi T., Ito S. (2018). Circulating levels of carbamylated protein and neutrophil extracellular traps are associated with periodontitis severity in patients with rheumatoid arthritis: a pilot case-control study. *PLoS One*.

[95] Konig M. F., Abusleme L., Reinholdt J. (2016). Aggregatibacter actinomycetemcomitans-induced hypercitrullination links periodontal infection to autoimmunity in rheumatoid arthritis. *Science Translational Medicine*.

[96] Hughes E. L., Becker F., Flower R. J., Buckingham J. C., Gavins F. N. E. (2017). Mast cells mediate early neutrophil recruitment and exhibit anti-inflammatory properties *via* the formyl peptide receptor 2/lipoxin A_4_ receptor. *British Journal of Pharmacology*.

[97] Headland S. E., Jones H. R., Norling L. V. (2015). Neutrophil-derived microvesicles enter cartilage and protect the joint in inflammatory arthritis. *Science Translational Medicine*.

[98] Singh J. A., Saag K. G., Bridges S. L. (2016). 2015 American College of Rheumatology Guideline for the treatment of rheumatoid arthritis. *Arthritis & Rhematology*.

[99] Burmester G. R., Rubbert-Roth A., Cantagrel A. (2014). A randomised, double-blind, parallel-group study of the safety and efficacy of subcutaneous tocilizumab versus intravenous tocilizumab in combination with traditional disease-modifying antirheumatic drugs in patients with moderate to severe rheumatoid arthritis (SUMMACTA study). *Annals of the Rheumatic Diseases*.

[100] Garley M., Jabloňská E., Suraźyński A. (2017). Cytokine network & NETs. *Folia Biologica*.

[101] Giaglis S., Stoikou M., Sur Chowdhury C. (2016). Multimodal regulation of NET formation in pregnancy: progesterone antagonizes the pro-NETotic effect of estrogen and G-CSF. *Frontiers in Immunology*.

[102] Ye L., Jiang B., Deng J. (2015). IL-37 alleviates rheumatoid arthritis by suppressing IL-17 and IL-17-triggering cytokine production and limiting Th17 cell proliferation. *Journal of Immunology*.

